# Disseminated BCGitis with Interferon-Gamma Receptor Deficiency: An Example of an Extremely Rare Illness

**DOI:** 10.7759/cureus.78654

**Published:** 2025-02-06

**Authors:** Ahmed Albishri, Eman J Ghazwani, Shady Wafa, Ali S Alquraishi, Badriah G Alasmari, Sami E Abdelmogeit, Jameelah A Alqahtani, Mohammed M Almusdi

**Affiliations:** 1 Department of Pediatrics, Pediatrics Infecious Disease, Armed Forces Hospital Southern Region, Khamis Mushait, SAU; 2 Department of Pediatrics, Armed Forces Hospital Southern Region, Khamis Mushait, SAU; 3 Department of Pediatrics, Endocrinology Unit, Armed Forces Hospital Southern Region, Khamis Mushait, SAU; 4 Department of Pediatric Medicine, Armed Forces Hospital Southern Region, Khamis Mushait, SAU; 5 Pediatric Intensive Care Unit, Khamis Mushait Maternity and Children Hospital, Khamis Mushait, SAU

**Keywords:** disseminated bcgitis, ifngr1, ksa, mycobacteriosis immunodeficiency, primary immunodeficiency

## Abstract

Mendelian susceptibility to mycobacterial disease (MSMD) is a group of inherited inborn errors of immunity due to approximately 21 genetic defects. Interferon-gamma receptor type 1 (IFNGR1) deficiency was the first disease described in this group. IFNGR1 can cause a loss of cellular responsiveness to interferon-γ (IFN-γ). Mycobacterial infections occur due to gene mutations that encode the IFNGR1 chain, leading to a loss of cellular responsiveness to type II IFN-γ, which plays a significant role in controlling intracellular bacteria. MSMD is characterized by increased susceptibility to environmental mycobacteria and low virulent mycobacteria like Bacillus Calmette-Guerin (BCG) vaccine strains. Careful and timely interventions for diagnosis and management are required if a patient develops clinical manifestations after BCG vaccination. Diagnosis can be made by gene study, and bone marrow transplantation remains the mainstay of treatment.

## Introduction

Primary immunodeficiency (PID) is a group of conditions in which some parts of the immune system are missing or not functioning. They can be presented clinically by increasing risk of infection, autoimmune and autoinflammatory diseases, allergies, malignancy, and bone marrow failure. PID can be classified as many inherited disorders, often due to single-gene mutations, that result in the specific impairment of normal immune development and function [1].

In 2022, the International Union of Immunological Society Expert Committee updated the classification of the inborn errors of immunity due to defects of intrinsic and innate immunity, and mendelian susceptibility to mycobacterial diseases (MSMD) is one of these errors of immunity. This has been reported in patients who received the Bacillus Calmette-Guerin (BCG) vaccine at birth and then developed disseminated infection [2]. In 1996, the first genetic etiology of MSMD was discovered, and the first deficiency to be identified among the other causes was interferon-gamma receptor type 1 (IFNGR1) deficiency [3]. IFNGR1 deficiency is associated with immunodeficiency 27 A and 27B, which leads to an increased risk of mycobacterial infections. MSMD can be inherited by autosomal dominant (AD) (27 B IFNGR1 deficiency), X-linked recessive (XR), or autosomal recessive (AR) (27 A IFNGR1) patterns. All of them can be partial deficiency or complete deficiency based on residual response to stimulation of receptors [4].

*IFNGR1* gene mutations can cause two phenotypes, complete or partial signaling defects. Complete signaling defects present with lepromatoid-like granulomas (diffuse and poorly differentiated) with many bacilli. Its prognosis is poor and can be treated only with bone marrow transplantation (BMT). Complete signaling defects have two subgroups, and both show an AR inheritance. Partial signaling defects caused by mutations in the *IFNGR1* gene may be inherited either as an AR trait or as an AD trait. Partial signaling defects have a better prognosis and can be treated with IFN-γ or antimycobacterial chemotherapy [5]. Partial IFNGR1 deficiency can be managed by IFN-γ replacement therapy but it is not effective in patients with complete deficiency due to the complete defect in IFNGR expression, so the mainstay of treatment in these patients is hematopoietic stem cell transplantation (HSCT).

Here, we report a case of a male patient from the southern region of Saudi Arabia with partial signaling defect (AD) mycobacteriosis immunodeficiency 27B, who presented with low-grade, continuous fever for three weeks with enlarged lymph nodes, and splenomegaly after receiving the BCG vaccine. Lymph node biopsy was sent for histopathology and showed chronic granulomatous inflammation, multinucleated giant cells with focal necrosis, and abscess formation. Also, polymerase chain reaction (PCR) for *Mycobacterium tuberculosis* (GeneXpert; Cepheid, Sunnyvale, California, United States) was positive. Confirmation of the diagnosis was done by genetic testing. 

## Case presentation

A seven-year-old male child, who was medically free till the age of four months, developed a low-grade, continuous fever for three weeks with an enlarged lymph node around 4x3 cm in size in the left axilla followed by maculopapular rash, non-blanching, in the face, chest and abdomen, and splenomegaly. He had received the BCG vaccine after birth without complications. There was no history of other systems' affection.

The patient was admitted to the hospital for a workup for a fever of unknown origin and a full lab investigation was done with supportive measures. The patient clinically improved but continued to have lymphadenopathy and splenomegaly. Peripheral blood smear showed toxic neutrophil granulation with eosinophilia and also in bone marrow aspiration.

**Figure 1:**
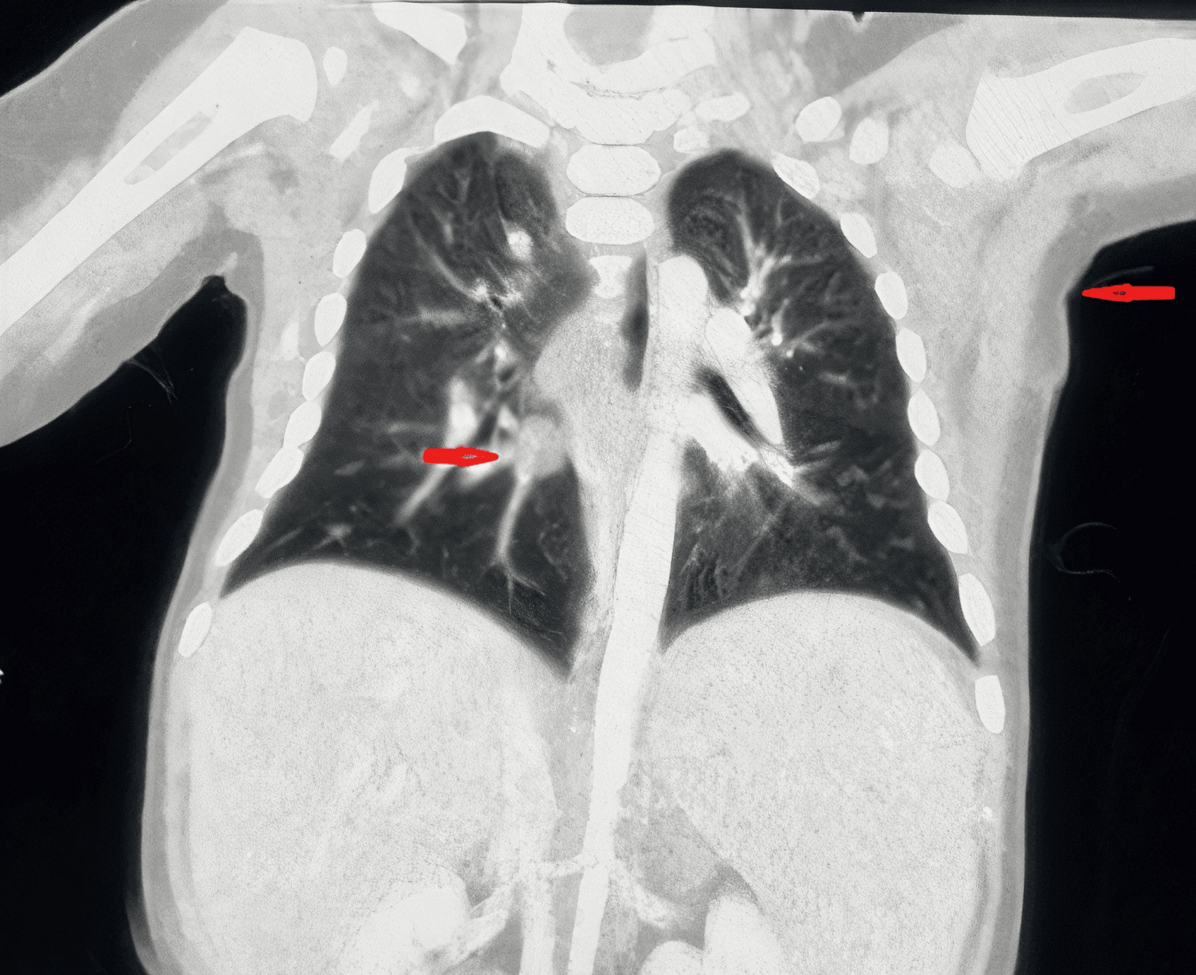
CT chest (sagittal view) showing left axillary ill-defined mass lesion with middle lobe small pulmonary nodule

A left axillary lymph node biopsy was sent for histopathology and the result came with chronic granulomatous inflammation having small clusters of epitheloid histocytes, multinucleated giant cells with focal necrosis, and abscess formation. Also, PCR for *M. tuberculosis* was positive. Computerized tomography (CT) chest and abdomen was done and the chest showed a left axillary ill-defined mass lesion about 2.5x2.5 cm (Figure 1), a sub-carinal necrotic lymph node with a middle lobe small pulmonary nodule. CT abdomen showed an enlarged liver and spleen with splenic small focal lesions. CT neck was done and showed multiple bilateral reactive deep cervical lymph nodes with reactive left submandibular lymph nodes.

Periodic acid-Schiff (PAS) and Ziehl-Nelsen (ZN) stains came positive for acid-fast bacilli (AFB). So the patient received antituberculous medication rifampicin, pyrazinamide, ethambutol, isonicotinic acid hydrazide (INH), and pyridoxine. The patient was discharged in good condition and continued his medication for one year and six months. The primary diagnosis was tuberculous lymphadenitis to rule out immunodeficiency; whole exome sequencing (WES) was sent and the result came later with AD mycobacteriosis immunodeficiency 27B.

**Figure 2:**
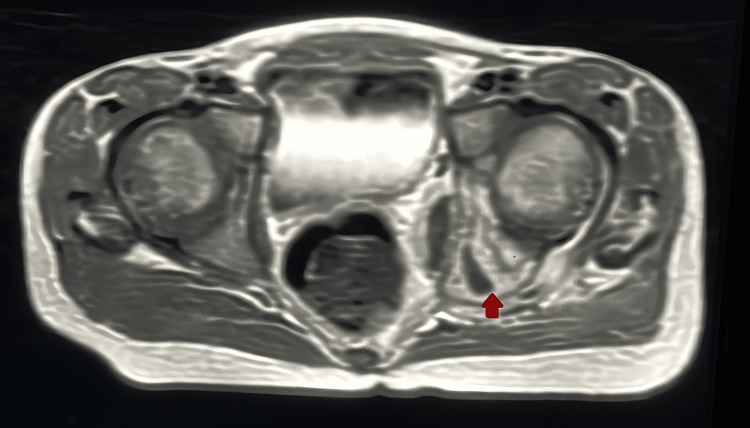
MRI showing sizable collection at the hip joint

**Figure 3:**
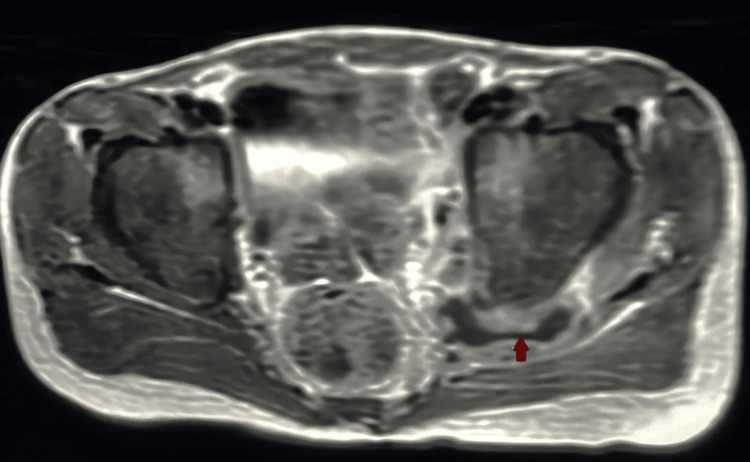
MRI showing enlarged left side pelvic floor muscles with small irregular abscess

At the age of six years, the patient presented at our ER complaining of fever and left hip joint pain with limping and limitation of movement for two weeks with no history of trauma. The patient was admitted to our hospital under the Orthopedic team and initially, a hip joint ultrasound was done that showed a moderate amount of joint effusion. This was followed by a hip joint magnetic resonance imaging (MRI) for more evaluation which showed a sizable collection at the hip joint (Figure 2) at the acetabulum and lateral part of pubic bone edema with enlarged left side pelvic floor muscles with small irregular abscess (Figure 3), along with a small rounded area of abnormal intensity in the proximal left femur. These findings suggested septic arthritis.

Laboratory investigation was done and inflammatory markers were very high. Based on these findings, the patient underwent incision, debridement, and drainage of the abscess, and the sample was sent for culture and sensitivity, which came positive for AFB *Mycobacterium avium*. The Infectious Disease team was informed and started antituberculous medication (INH, pyrazinamide, ethambutol, pyridoxine). After stabilization and improvement, the patient was discharged with the advice to continue medication. After one month, the MRI was repeated and showed moderate improvement with regard to reduced size and number of previously seen multiple locations in the region of the left hip joint with inflammatory changes still noted in tissues around the left joint and swollen left internal obturator muscle with a tiny abscess inside. The patient received oral anti-tuberculus medication for one year followed by clarithromycin as prophylaxis. The patient is following up with the Infectious Disease team regularly and is doing well. 

## Discussion

IFNGR1 deficiency was the first mutation to be identified among the causes of MSMD [3]. IFNGR1 deficiency is associated with immunodeficiency 27A and 27B, increasing the risk of mycobacterial infections and infections by intracellular microorganisms, viruses, *Toxoplasma*, and *Histoplasma*. MSMD can be inherited by AR or AD patterns [4]. In our report, we described a male patient with AD mycobacteriosis immunodeficiency 27B who presented to our hospital initially at the age of four months with prolonged low-grade fever, cervical and axillary lymphadenitis, and hepatosplenomegaly and managed initially as fever of unknown origin with all laboratory studies for suspected differential diagnosis as viral infection with lymphadenites like Epstein-Barr virus (EBV) and cytomegalovirus (CMV) and also bacterial infection with lymphadenitis like *Tuberculosis lymphadeni* as all have similar manifestations. Following all results, we strongly suspected tuberculous lymphadenitis due to immune deficiency, so WES was done that helped with the diagnosis. Again, at the age of six years, the patient came with fever and left hip joint septic arthritis due to AFB *M. avium*.

In a report of two patients from related consanguineous families with AR complete IFN-γ deficiency, the first patient received their vaccination with BCG at the age of three months, and three weeks later, she developed a large left axillary mass, hepatosplenomegaly, and maculopapular rashes in association with prolonged fever [6]. A lymph node biopsy was excised for AFB and came positive, confirming the diagnosis of disseminated mycobacterial disease and treated with antituberculous medications. The second patient received his BCG vaccine at the age of three months and then six weeks after the initial vaccination presented with the same presentation, and a biopsy of an excised lymph node confirmed the diagnosis of disseminated mycobacterial disease and treated with antituberculous medications [6]. Jouanguy et al. reported a female child aged 2.5 months who developed fever, diffuse pneumonitis, hepatosplenomegaly, regional adenitis, lymph node enlargement, and multiple osteolytic lesions [7]. All these manifestations appeared after BCG vaccination and one month later, a mycobacterium species was cultured from the lungs and bone marrow and identified as *Mycobacterium bovis* BCG strain and finally diagnosed as IFNGR1 deficiency, the same diagnosis as our patient. In another report by Esmaeilzadeh et al., their patient was a 19-month-old female child who presented with a history of fever for 14 days [8]. Flow cytometry was done and came near normal; IgM and IgE were high. Chest X-ray showed right hilar pneumonic infiltration and para-aortic lymphadenopathy. *Aspergillus fumigatus *PCR was positive. WES study showed *SH2B3* and *IFNGR1* mutations, indicating that patients with IFNGR1 deficiency can be presented with aspergillosis.

This case helps to increase awareness of the occurrence of IFNGR1 deficiency, which is a very rare subtype of MSMD. It spotlights the importance of early diagnosis of this rare association, potentially enabling early interventions to improve clinical outcomes. 

## Conclusions

IFNGR1 deficiency is a very rare subtype of MSMD that makes the patient prone to mycobacterial infections and intracellular microorganisms. It should be suspected if a patient develops clinical manifestations after BCG vaccination. Partial signaling defects have a better prognosis than complete signaling defects and treatment with IFN-γ or antimycobacterial chemotherapy could be done. In contrast, complete signaling defects prognosis is poor and treatment can be done only with BMT.
